# Correction: *In vitro* toxicity evaluation of silica-coated iron oxide nanoparticles in human SHSY5Y neuronal cells

**DOI:** 10.1039/c6tx90013e

**Published:** 2016-05-05

**Authors:** Gözde Kiliç, Carla Costa, Natalia Fernández-Bertólez, Eduardo Pásaro, João Paulo Teixeira, Blanca Laffon, Vanessa Valdiglesias

**Affiliations:** a DICOMOSA Group , Department of Psychology , Area of Psychobiology , Universidade da Coruña , Research Services Building , Campus Elviña s/n , 15071-A Coruña , Spain . Email: blaffon@udc.es ; Fax: +34 981167172 ; Tel: +34 981167000; b Department of Cell and Molecular Biology , University of A Coruña , Faculty of Sciences , Campus A Zapateira s/n , 15071-A Coruña , Spain; c Department of Environmental Health , Portuguese National Institute of Health , Rua Alexandre Herculano 321 , Porto 4000-055 , Portugal; d EPIUnit - Institute of Public Health , University of Porto , Rua das Taipas no. 135 , Porto 4050-600 , Portugal

## Abstract

Correction for ‘*In vitro* toxicity evaluation of silica-coated iron oxide nanoparticles in human SHSY5Y neuronal cells’ by Gözde Kiliç *et al.*, *Toxicol. Res.*, 2016, **5**, 235–247.



## 

Fig. 10 is incorrectly published with asterisks missing. The correct Fig. 10 is below:


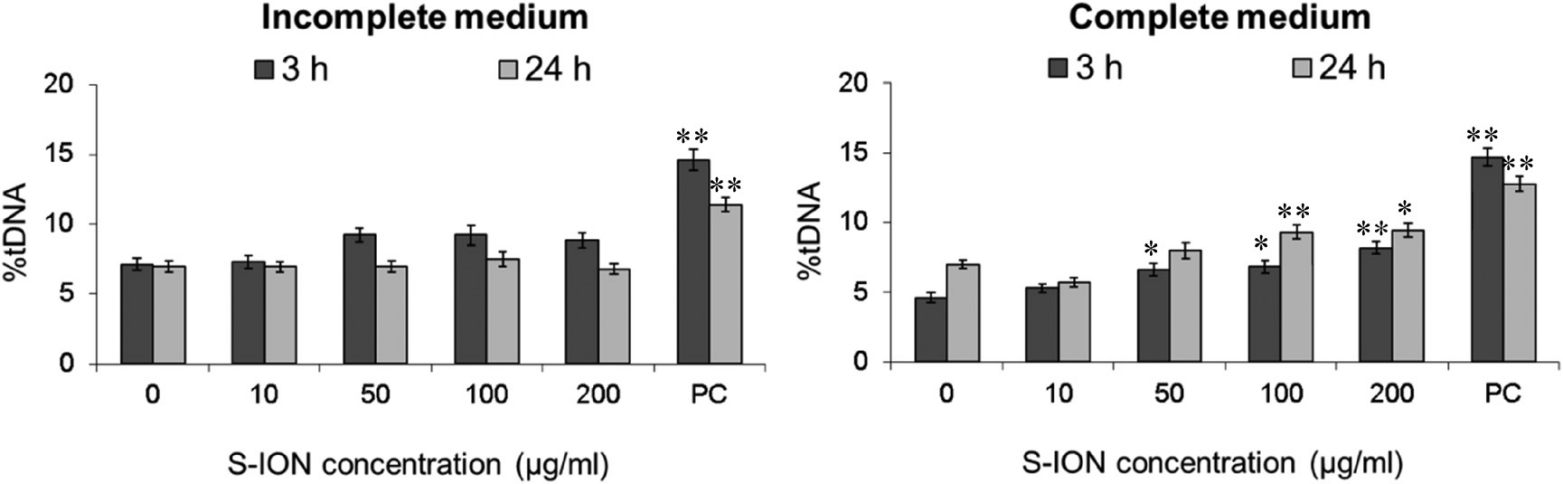
The Royal Society of Chemistry apologises for these errors and any consequent inconvenience to authors and readers.

